# The beneficial effects of traditional Chinese medicine on antioxidative status and inflammatory cytokines expression in the liver of piglets

**DOI:** 10.3389/fvets.2022.937745

**Published:** 2022-09-23

**Authors:** Xiaoyu Wang, Yun Wang, Yaqin Mao, Aiming Hu, Tianfang Xu, Yan Yang, Feibing Wang, Guangbin Zhou, Xiaowang Guo, Huabin Cao, Fan Yang

**Affiliations:** ^1^Jiangxi Provincial Key Laboratory for Animal Health, Institute of Animal Population Health, College of Animal Science and Technology, Jiangxi Agricultural University, Nanchang, China; ^2^Department of Animal Science and Technology, Jiangxi Biotech Vocational College, Nanchang, China; ^3^China Institute of Veterinary Drug Control, MOA Center for Veterinary Drug Evaluation, Beijing, China; ^4^Jian City Livestock and Veterinary Bureau, Ji'an, China; ^5^Jiangxi Agricultural Technology Extension Center, Nanchang, China; ^6^Agricultural Technology Extension Center, Jinxi County Agriculture and Rural Bureau, Fuzhou, China; ^7^Animal Epidemic Prevention and Quarantine Unit, Fengcheng Agricultural and Rural Bureau, Fengcheng, China; ^8^Yichun Agriculture and Rural Affairs Bureau, Yichun, China

**Keywords:** traditional Chinese medicine, piglet, antioxidant capability, inflammation, liver

## Abstract

Oxidative stress and inflammation seriously affected the growth and development of piglets. Traditional Chinese medicine (TCM) prescriptions has been used to prevent various diseases of piglets, including anti-inflammatory and antioxidant. Here, we identified the effects of Xiao-Jian-Zhong-Tang (XJZT) and Jingsananli-sepsis (JJS) on the oxidative stress and inflammatory in the liver of piglets. The piglets were fed with the basal diet (Control group), basal diet affixed with 10 g/kg XJZT (TCM I group), and basal diet affixed with 3 g/kg JJS (TCM II group), respectively. The serum was gathered on days 30 and 60 and the liver samples were also collected on day 60. Results showed that the TCM I and TCM II markedly increased the activities of the glutathione peroxidase (GSH-Px) and total antioxidant capacity (T-AOC), and reduced the levels of malonaldehyde (MDA), TNF-α, IL-6, and IL-8 in serum. In addition, compared to the control group, Nrf2, SOD-1, NQO-1, and HO-1 mRNA expression levels and the protein levels of Nrf2 and HO-1 were significantly increased while NF-κB, TNF-α, IL-6, and IL-8 mRNA expression levels and the phosphorylation levels of NF-κB and IκB-α were decreased in TCM I and TCM II groups. Collectively, these findings suggested that TCM I and TCM II could enhance anti-oxidative and anti-inflammatory capabilities in the liver of piglets via the Nrf2/NF-κB pathway, providing a basis for the functional exploration of TCM prescriptions.

## Introduction

The climbing requirement for animal products in recent decades has led to the development of intensive animal production systems which has been demonstrated to produce stress responses in animals (1). Most human practices toward animals could lead to stress responses like climate change, social, environmental, and immunological stress, thereby causing oxidative stress (2–4). In consideration of global climate change, heat stress as a kind of oxidative stress brings about hundreds of millions of dollars of economic losses in the swine industry annually (5). Therefore, combatting oxidative stress is absolutely crucial for the swine industry. Generally, there is an imbalance between the production of reactive oxygen species (ROS) and the biological ability to clear reactive intermediates (6). When out of control, the imbalance of ROS dynamic is hazardous to cellular macromolecules, posing toxic effects in the function and survival of numerous organs (7). Elevated levels of oxidative stress can impair milk production and reproductive performance of sows, which can affect not only the life of sows, but also the health of piglets (8). On the other hand, the occurrence of inflammation could be caused by increased ROS (9). Inflammation is an aspect of the immune response to injury and disease, whose process is closely related to the occurrence of oxidative stress (10). Thus, the two processes are crucial targets of developing therapeutics against numerous diseases.

Currently, traditional Chinese medicine (TCM) composed of natural plant derivative has been practiced to improve farm-animal health and prevent various diseases (11, 12). Compound preparations can potentially exert multiple effects in a distinct mechanism that is expected to reach more comprehensive effect by targeting multi pathways and multi targets (13). Xiao-Jian-Zhong-Tang (XJZT) consists of 10 medicinal herbs (Cassia Twig, Glycyrrhiza uralensis, Ziziphus zizyphus, Cynanchum otophyllum, Zingiber officinale Roscoe, Rhizoma Atractylodes, Atractylodes macrocephala, Poria cocos, Coptis chinensis Franch and Maltose) and is currently used to treat chronic liver diseases (14). Jingsananli-sepsis (JSS) composed of 8 medicinal herbs (Nepeta cataria L, Radix Saposhnikoviae, Notopterygium incisum, Radix Angelicae pubescentis, Radix bupleuri, Radix Peucedani, Poria cocos, and Glycyrrhiza uralensis), which have been used in the treatment of grippe, and fever with a long history (15). These may be related to their prescriptions composition consists with various types of antimicrobial, immunoregulatory, anti-oxidative and anti-inflammatory active substances (16, 17). Many functional components in these two of TCM, including flavonoids, volatile oils, polysaccharides and organic acids have been demonstrated that were closely related to the immunity enhancement. The flavonoids have antioxidant activity of reduction of free radical formation and free radical scavenging (18). Volatile oil, a major active compound of many herbs, is known to inhibit oxidative stress, and inflammation (19). Additionally, polysaccharides and organic acids are also widely used in inhibits oxidative stress, bacterium and virus (20, 21).

Nuclear factor E2 related factor 2 (Nrf2) acts as a main regulatory factor in preserving cellular defense against oxidative stress. Evidence have been provided that it is responsible for the protection of liver injury and inflammation caused by oxidative stress in the way of regulating antioxidant proteins expression levels (14, 22). On the other hand, many *in vivo* and *in vitro* experiments have demonstrated that oxidative stress could activate NF-κB pathway. Additionally, the activation of NF-κB is thought to be a response to oxidative stress. This signaling pathway is a master regulator of inflammation and may the target of the anti-inflammatory effect of TCM. TCM are usually used to counteract diseases. Whereas studies on the effects of TCM with respect to their anti-inflammatory and anti-oxidative response are rather scanty. Hence, this study explored the potential effects of TCM prescriptions (10 g/kg XJZT and 3g/kg JSS) as feed additives on antioxidative status and inflammatory reaction in piglets, to shed light on the functional role of XJZT and JSS prescriptions.

## Materials and methods

### Animal treatments

All experimental procedures were performed by Jiangxi Agricultural University Animal Care and Use Committee. The experiment lasted for 60 days, and a total of thirty crossbred (Durox × Landrace × Yorkshire) piglets (weight 21.43 ± 2.86 kg) were randomly allotted to three dietary treatments groups on average according to initial body weight. The dietary treatments were basal diet (control group), 10 g/kg Xiao-Jian-Zhong-Tang (XJZT) combination with basal diet (TCM I group) and 3 g/kg Jingsananli-sepsis combination with basal diet (TCM II group). The composition and nutrient levels of basal diet are shown in Table 1. All raw materials for TCM I were bought from Changsheng pharmacy (Jiangxi, China) and TCM II was provided by The Spirit Jinyu Biological Pharmaceutical Co., Ltd. (Huhhot, Inner Mongolia, China). All dried herbs are crushed through a 2.5 mm screen. Composition and main active constituents of TCM I and TCM II are presented in Table 2.

**Table 1:** Composition and nutrient levels of basal diet (air-dry basis).

**Ingredients**	**Content**	**Analyzed**	**Content**
	**(%)**	**composition, g/kg**	**(%)**
Maize	55.80	DM	89.21
Soybean meal	16.30	DE^2^ (MJ/kg)	14.36
Fermented soybean meal	7.00	Crude Protein (CP, %)	19.63
Wheat middling	4.50	Lysine	1.32
Fish meal	2.50	Methionine	0.43
Dried porcine solubles	2.50	Methionine + Cystine	0.77
Whey powder	6.25	Threonine	0.81
Soy oil	1.65	Calcium	0.96
Lysine	0.25	Total phosphorus	0.60
Methionine	0.10	Total	100.00
Limestone	1.05		
CaHPH_4_	0.80		
NaCl	0.30		
Vitamin-mineral premix^1^	1.00		
Total	100.00		

1 The premix provides following per kilogram diet: Vitamin A 8 000 IU, Vitamin D 2 500 IU, Vitamin E 15 mg, nicotinic acid 20 mg, D-pantothenie 10 mg, riboflavin 4 mg, biotin 0.06 mg, folic acid 0.2 mg, thiamine 2 mg, choline chloride 500 mg, copper 165 mg, iron 110 mg, manganese 80 mg, zinc 330 mg, selenium 0.20 mg.

^2^Digestible energy is calculated value according to ingredients energy, the others are measured values.

**Table 2:** Composition and main active constituents of TCM I and TCM II (air dry basis)^1^.

**Latin name**	**Main active**	**Used part**	**Content**
	**constituent**		**(%)**
**TCM I**			
Cassia Twig	Cinnamaldehyde	Dried twig	13.0
Glycyrrhiza uralensis	Glycyrrhizin	Dried root	4.0
Ziziphus zizyphus	Jujuba polysaccharide	Dried fructification	4.0
Cynanchum otophyllum	Paeoniflorin	Dried root	13.0
Zingiber officinale Roscoe	Ginger oleoresin	Dried root	6.0
Rhizoma atractylodes	Atractylodine	Dried root	14.0
Atractylodes macrocephala	Biatractylolide	Dried root	10.5
Poria cocos	Pachymaran	Dried sclerotium	10.5
Coptis chinensis Franch.	Berberine	Dried root	4.0
Maltose	Maltose	-	21.0
Total			100.0
**TCM II**			
Nepeta cataria L.	Nepeta Cataria Oil	Dried stem	16.5
Radix Saposhnikoviae	Chromone glycoside	Dried root	16.5
Notopterygium incisum	Notopterol	Dried root and stem	16.5
Radix Angelicae pubescentis	Heraclenin	Dried root	16.5
Radix bupleuri	Saikosaponin	Dried root	10.0
Radix Peucedani	Peucedanin	Dried root	10.0
Poria cocos	Pachymaran	Dried sclerotium	10.0
Glycyrrhiza uralensis	Glycyrrhizin	Dried root	4.0
Total			100.0

^1^Main active constituents of TCM come from Chinese pharmacopeia (2005).

### Sample collection

Blood was obtained from the jugular vein in the collection tube on days 30 and 60. Serum was separated and stored at −20°C to detect the antioxidant indices and inflammatory cytokines. On the day 60 of the experiment, all piglets in each group were euthanized with sodium pentobarbital (40 mg/kg body weight). Livers were dissected immediately from all piglets. The blood of the liver was washed by precooled normal saline (0.9% NaCl, Beyotime, China), and the surface liquid was removed with filter paper, and then stored at −80°C for analysis after rapidly frozen in liquid nitrogen.

### Determination of antioxidant indices and inflammatory cytokines in serum

The levels of T-AOC, SOD, GSH-PX, and MDA in serum were measured strictly based on the instructions of the kits (Nanjing Jiancheng Bioengineering Institute, China; T-AOC, A015-2-1; SOD, A001-3-1; GSH-PX, A005-1-2; MDA, A003-1-2). The levels of TNF-α, IL-6, IL-8, and IL-10 in serum were measured by enzyme linked immunosorbent assay kits (R&D, USA; TNF-α, MTA00B; IL-6, D6050; IL-8, D8000C; IL-10, M1000B) according to the instructions. The optical density of each well was read at 450 nm with an Absorbance Microplate Reader (SpectraMax, China).

### Quantitative real-time PCR analysis

RT-qPCR assay was consistent with the method previously (23, 24). The primer sequences of Nrf2, SOD-1, NQO-1, HO-1, NF-κB, TNF-α, IL-6, IL-8, IL-10, and GAPDH are shown in Table 3.

**Table 3:** Primers used in this study.

**Gene**	**GeneBank Number**	**Primers Sequences (5^′^-3^′^)**	**AAnnealing** **temp (°C)**	**Amplification** **size (bp)**
Nrf2	XM_005671982.1	F: CCCATTCACAAAAGACAAACATTC R: GCTTTTGCCCTTAGCTCATCTC	58	72
SOD-1	NM_001190422.1	F: GAGACCTGGGCAATGTGACT R: CTGCCCAAGTCATCTGGTT	57	139
NQO1	NM_001159613.1	F: CCAGCAGCCCGGCCAATCTG R: AGGTCCGACACGGCGACCTC	66	160
HO-1	NM_001004027	F: CGCTCCCGAATGAACAC R: GCTCCTGCACCTCCTC	55	112
NF-κB	NM_001048232.1	F: CTCGCACAAGGAGACATGAA	58	147
		R: ACTCAGCCGGAAGGCATTAT		
TNF-α	NM_214022.1	F: CCAATGGGCAGAGTGGGTATG	56	117
		R: TGAAGAGGACCTGGGAGTAG		
IL-6	NM_001252429.1	F: TGGCTACTGCCTTCCCTACC	58	132
		R: CAGAGATTTTGCCGAGGATG		
IL-8	NM_213867.1	F: TTCGATGCCAGTGCATAAATA	57	176
		R: CTGTACAACCTTCTGCACCCA		
IL-10	NM_214041	F: CGGCGCTGTCATCAATTTCTG	58	89
		R: CCCCTCTCTTGGAGCTTGCTA		
GAPDH	NM_001206359	F: ACTCACTCTTCCACTTTTGATGCT R: TGTTGCTGTAGCCAAATTCA	57	100

F, Forward primer; R, Reverse Primer.

### Western blot analysis

Western blotting assay was performed in accordance with the procedure in a previous study (Dai et al., 2021). The primary antibodies were Nrf2 (Proteintech, 1:1000), HO-1 (Wanleibio, 1:1000), NF-κB P65 (Bioss, 1:1000), phospho-NF-κB (Bioss, 1:1000), IκB-α (Wanleibio, 1:1000), phospho-IκB-α (Bioss, 1:1000), and GAPDH (1:8000; Bioss, China).

### Statistical analysis

Quantitative variables are expressed as the mean ± standard deviation (SD). All statistical analyses were calculated by one-way analysis of variance and the least significant difference test. *P* values < 0.05 was considered significant.

## Results

### Effects of TCM I and TCM II on antioxidant capabilities in serum

**Figure 1:**
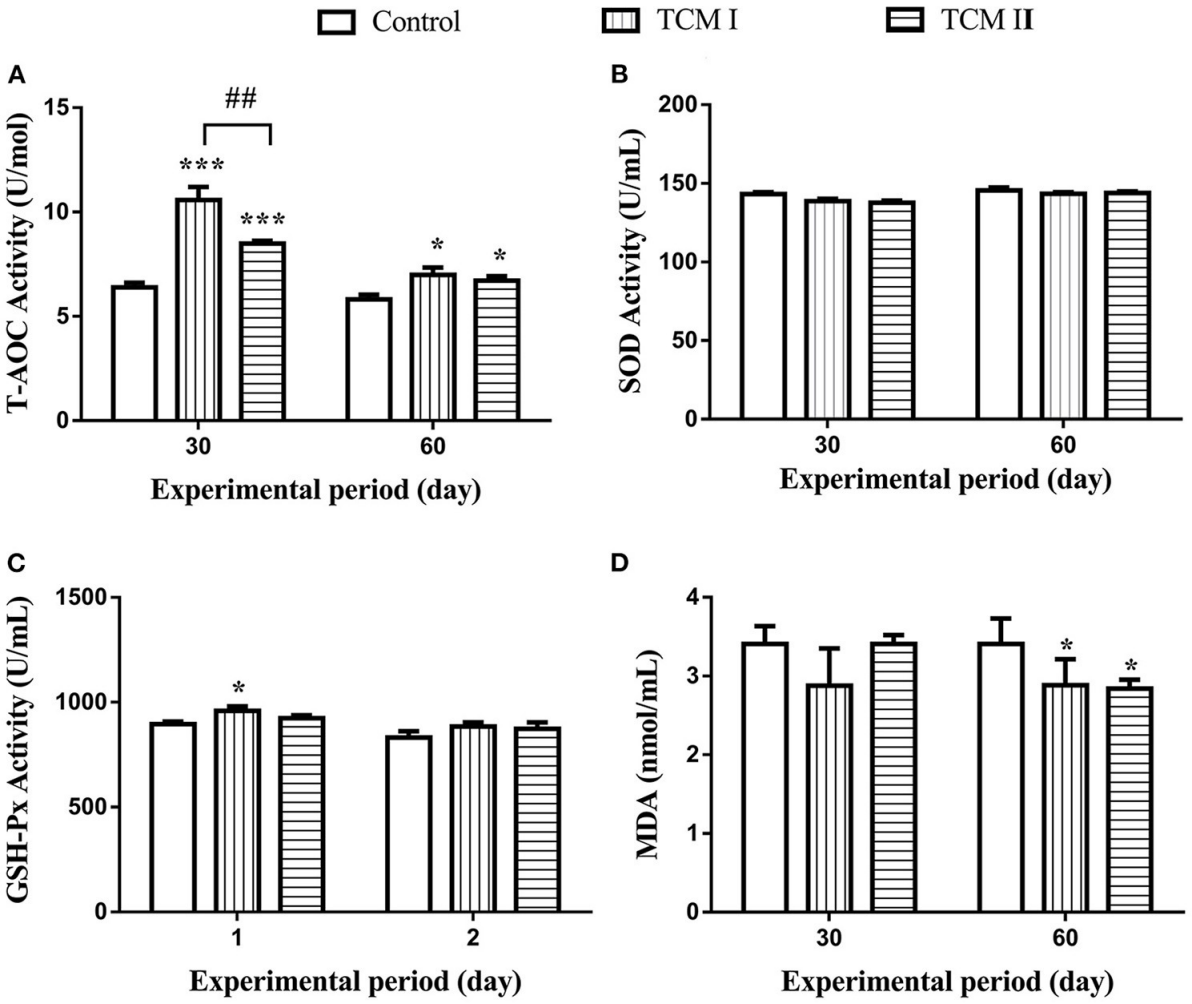
Determination of **(A)** T-AOC, **(B)** SOD, **(C)** GSH-Px, and **(D)** MDA levels in serum on days 30 and 60. The data are presented as the mean ± SD of at least three independent experiments (*n* ≥ 3). “*” indicates a significant difference compared with control group (*P < 0.05, **P < 0.01, and ***P < 0.001). “^#^” indicates a significant difference between the indicated groups (^#^*P* < 0.05, ^##^*P* < 0.01, and ^###^*P* < 0.001). The same scheme also applies to the remaining figures.

As shown in Figures 1A,B, T-AOC level in TCM I and TCM II groups was obviously higher than the control group (*P* < 0.05 or *P* < 0.001) on days 30 and 60. In addition, GSH-Px level was notably increased (*P* < 0.05) in TCM I group compared to the control group (Figure 1C). Furthermore, the T-AOC activity in TCM I and TCM II groups was markedly upregulated (*P* < 0.05) compared to the control group on day 60. However, MDA content was significantly decreased (*P* < 0.05) in TCM I and TCM II groups compared to the control group on day 60 (Figure 1D).

### Effects of TCM I and TCM II on inflammatory cytokines in serum

**Figure 2:**
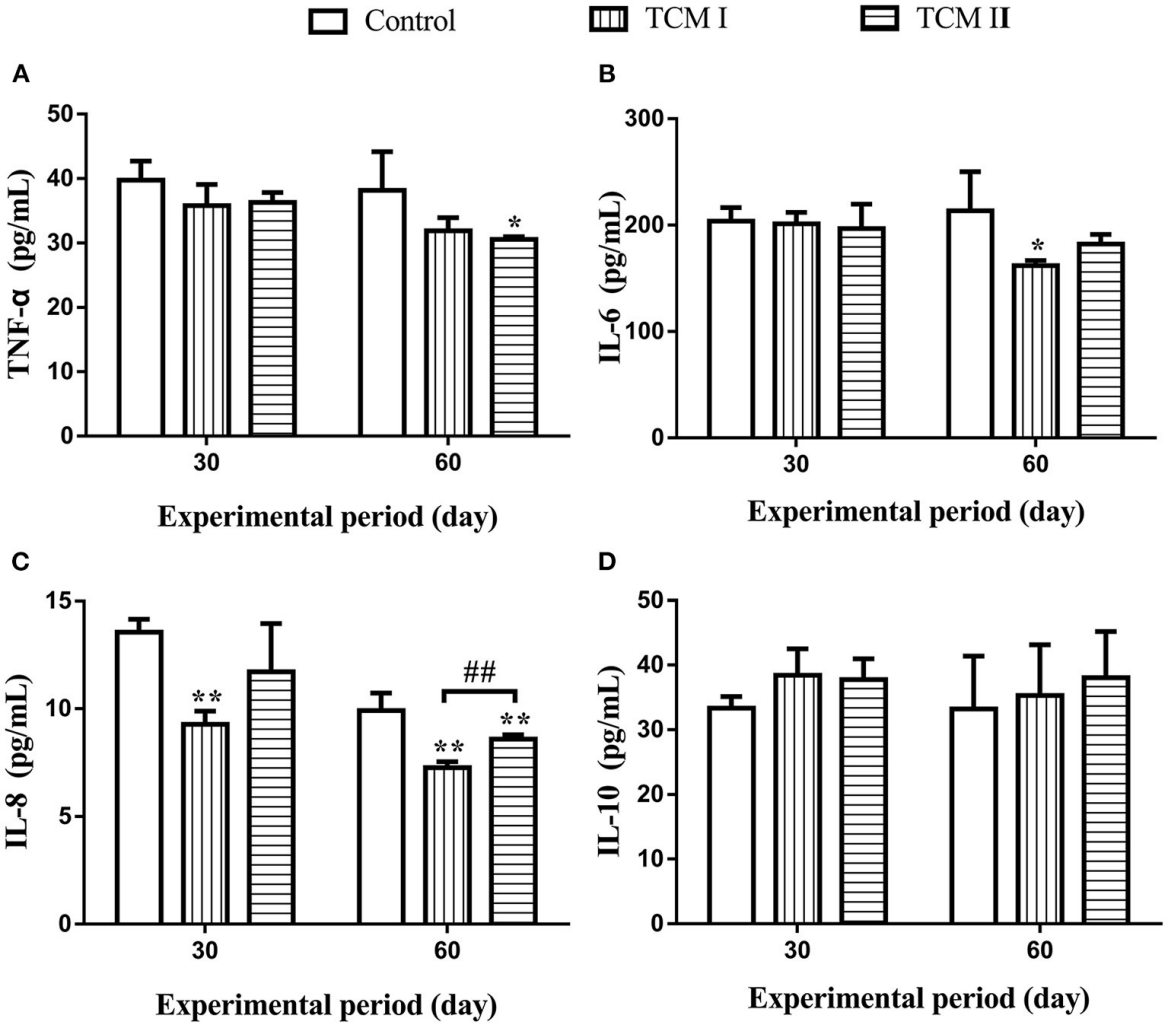
Determination of **(A)** TNF-α, **(B)** IL-6, **(C)** IL-8, and **(D)** IL-10 levels in serum on days 30 and 60.

As shown in Figures 2A–C, TNF-α, IL-6 and IL-8 levels in the TCM I and TCM II groups were significantly lower than the control group (*P* < 0.05 or *P* < 0.01) on days 30 and 60. Moreover, IL-8 level was markedly decreased (*P* < 0.01) in the TCM I group compared to the TCM II group on day 60. Additionally, IL-10 level was increased in TCM I and TCM II groups in comparison with the control group (Figure 2D).

### Effects of TCM I and TCM II on mRNA levels of antioxidant-related genes and protein levels in liver

**Figure 3:**
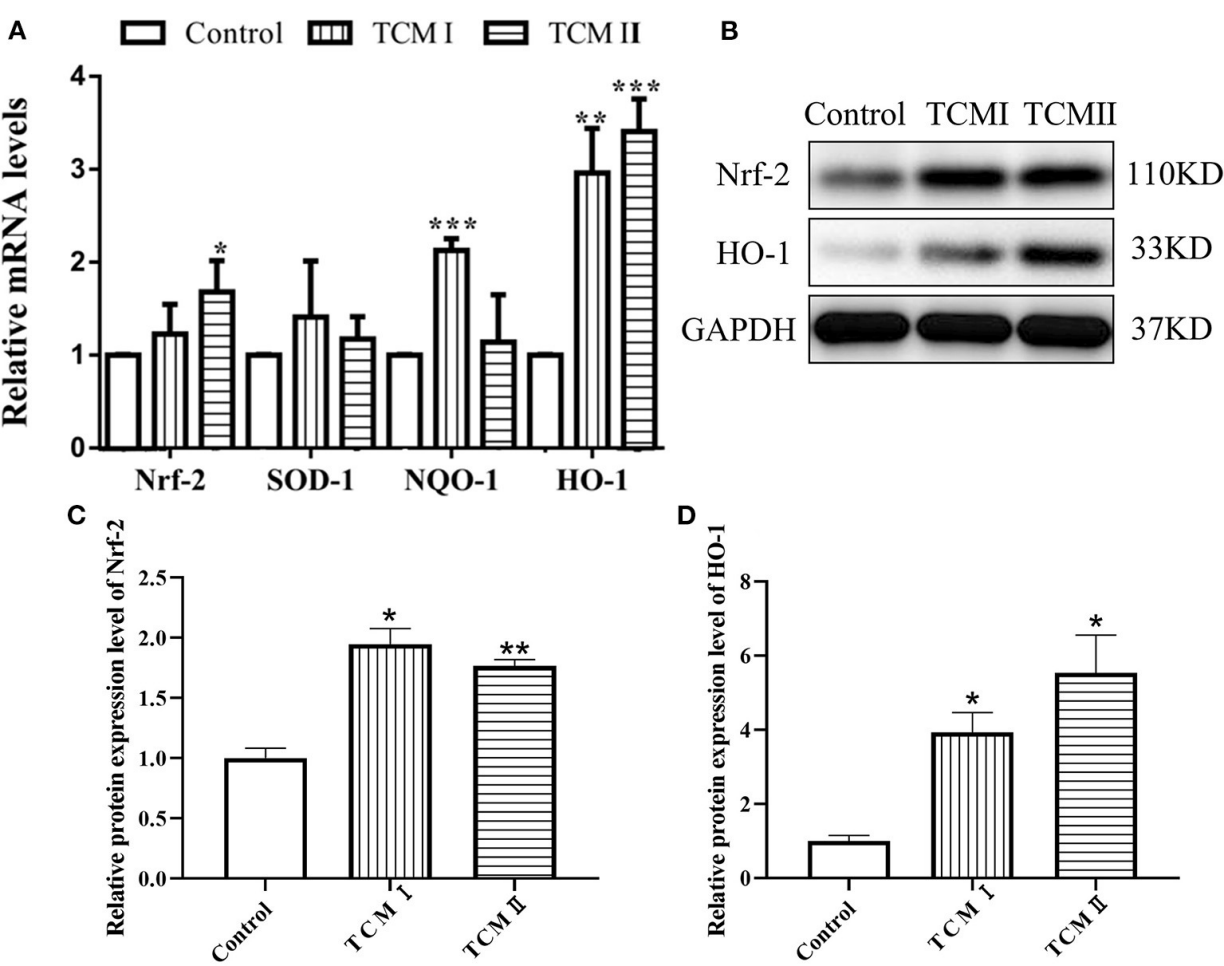
Effects of TCM I and TCM II on the mRNA levels of antioxidant-related genes and protein levels in liver. **(A)** mRNA levels of Nrf-2, SOD-1, NQO-1, and HO-1. **(B)** Protein levels of Nrf-2 and HO-1. **(C)** Graph showing the protein level of Nrf-2. **(D)** Graph showing the protein levels of HO-1.

As described in Figure 3A, HO-1 and NQO-1 mRNA levels of liver in TCM and TCM II groups were higher than that in control group (*P* < 0.05 or *P* < 0.01). Otherwise, the mRNA level of SOD-1 was increased in TCM I and TCM II groups compared to the control group, whereas the differences were not significant. The protein levels of Nrf2 and HO-1 are presented in Figures 3B–D. Both Nrf2 and HO-1 protein levels in TCM I and TCM II groups were dramatically increased in comparison with the control group (*P* < 0.05 or *P* < 0.01).

### Effects of TCM I and TCM II on mRNA levels of inflammatory cytokines and protein levels in liver

**Figure 4:**
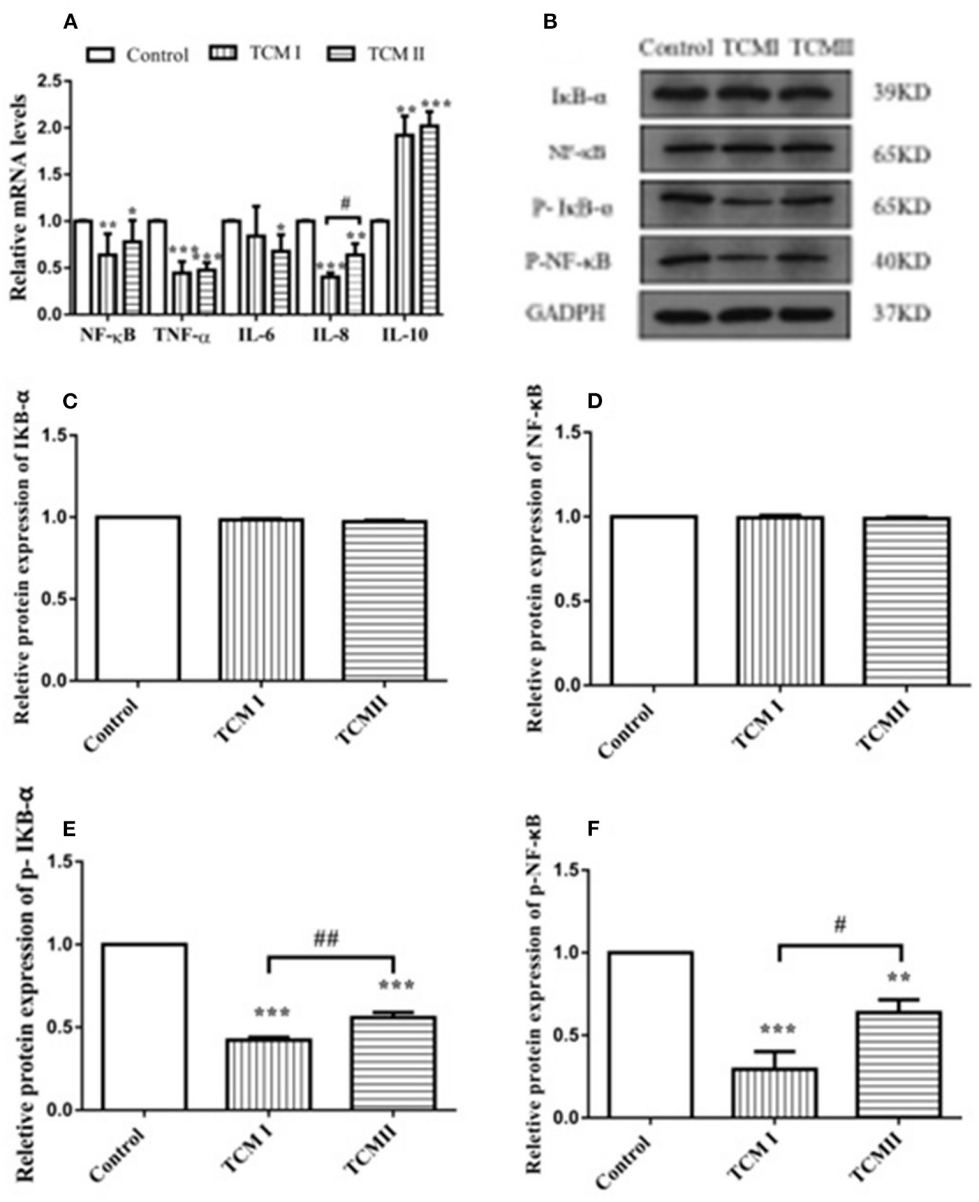
Effects of TCM I and TCM II on the mRNA levels of inflammatory cytokines and protein levels in liver. **(A)** mRNA levels of NF-κB, TNF-α, IL-6, IL-8, and IL-10. **(B)** Protein levels of IκB-α, NF-κB p65, p-IκB-α, and p-NF-κB. **(C)** Graph showing the protein level of IκB-α. **(D)** Graph showing the protein level of NF-κB p65. **(E)** Graph showing the protein level of p-IκB-α. **(F)** Graph showing the protein level of p-NF-κB.

As shown in Figure 4A, NF-κB, IL-8, and TNF-α mRNA levels in TCM I group were significantly downregulated compared to control group (*P* < 0.05 or *P* < 0.01), while IL-10 mRNA level was significantly increased (*P* < 0.01). Additionally, Figure 4A shows that NF-κB and IL-6 mRNA levels in the TCM II group also dramatically declined in comparison with control group (*P* < 0.01 or *P* < 0.001). However, compared to the control group, IL-10 mRNA level was markedly up-regulated in TCM II group (*P* < 0.001). The protein expressions of IκB-α and NF-κB had no significant differences in TCM I and TCM II groups (*P* > 0.05) in comparison with the control group (Figures 4B,C). As shown in Figures 4E,F the protein expressions of the p-IκB-α and p-NF-κB in TCM I and TCM II groups were dramatically downregulated in comparison with the control group (*P* < 0.01 or *P* < 0.001).

## Discussion

In intensive farming systems, piglets face numerous challenges like change in the nutritional source, feed contamination with mycotoxins, pathogenic micro-organisms, and some chemical agents, leading to excessive ROS accumulation that cause the occurrence of oxidative stress and inflammation (3, 25, 26). Accordingly, it is necessary to find functional products that can effectively protect piglets from oxidative stress and inflammation. Chinese herbal medicine is a unique medical resource in China. In most condition, many Chinese herbal medicines share the advantages of low toxicity, small side effects, low drug resistance and no residual, etc. (27, 28). Their application in livestock and poultry production not only enhanced the immune function of the body and improve disease resistance, but also promote the growth and development of animals. Due to the complicated process of oxidative stress and inflammation, the therapeutic effect of a single herb may not be ideal. XJZT and JSS are well-known traditional herbal medicines which have been used to alleviate oxidative stress and inflammation and improve immunity for a long time, containing with multiple antimicrobial, immunoregulatory, anti-oxidative and anti-inflammatory active substances (29–31). The flavonoids as a powerful antioxidant can scavenge free radicals from multiple targets, and they are safer and more effective than other antioxidants (19, 32). Additionally, polysaccharides and organic acids have abundant biological activities, so they can also widely used in inhibits oxidative stress and inflammation (20, 21). Therefore, this study investigated the effects of dietary supplementation of XJZT or JJS on the anti-oxidative capacity and inflammatory response in the liver of piglets.

Oxidative stress refers to a state of imbalance between oxidation and antioxidation in which numerous oxidized intermediates are produced. The impulse induction of protective antioxidant enzymes can exert profitable effects on the ability of the body to maintain homeostasis. T-AOC represents the enzymatic and non-enzymatic antioxidant defense systems. Superoxide anion radicals (O^2−^) can be scavenged by T-SOD and peroxides and hydroxyl radicals produced in the process of cell metabolism can be eliminated by GSH-Px, thereby protecting the body from oxidative damage (33, 34). Additionally, the degree of lipid peroxidation could be reflected by MDA content, so its level is proportional to the degree of cell damage (35). Previous reports have shown that subcutaneous lipopolysaccharide injection and supplement ampelopsin in diet for pigs improved the anti-oxidative capacity in plasma (36, 37). In this study, we found that supplementation with XJZT and JJS increased GSH-Px and T-AOC levels but decreased MDA content in plasma, indicating that XJZT and JJS supplementations in piglets could save tissues from lipid peroxidation. This benefit might be due to the reduction of the antioxidative system burden by dietary total flavonoids, polysaccharides, and phenolicsand. Additionally, changes in the levels of these antioxidant indexes (SOD, GSH-Px, and MDA) have affinities with Nrf2 expression level. Moreover, Nrf2 binds to antioxidant response elements such as HO-1 and NQO1 to prevent oxidative damage (38). Our results illustrated that XJZT and JJS could significantly increase Nrf2, SOD-1, HO-1, and NQO-1 mRNA levels and Nrf2 and HO-1 protein levels. These results demonstrated that XJZT and JJS against oxidative damage by activating the Nrf2 signal pathway in liver.

Additionally, there is a strong relationship between oxidative stress and inflammation. As a redox-sensitive transcription factor, NF-κB expression level can be promoted by oxidative stress, whose level can mediate the transcription of numerous inflammatory genes (39). TNF-α occupies an important position in the inflammatory responses which is responsible for plenty of cytokines and chemokines production (40, 41). IL-10 acts as an anti-inflammatory cytokine with important immunoregulatory functions in the way of restraining the inflammatory cytokines expression levels like TNF-α, IL-6 and IL-1 (40). In our study, the XJZT and JJS decreased the concentrations of TNF-α, IL-6, and IL-8 and increased IL-10 level in the plasma. NF-κB is of great importance for controlling the expression levels of inflammatory response related proteins. Under normal conditions, there is covalently binding between NF-κB and IκB in the cytoplasm. After receiving immune stimulation signals such as TNF-α and lipopolysaccharides, IκB is phosphorylated and degradated, followed by NF-κB translocation to the nucleus, promoting transcription of inflammatory genes like IL-1β, and IL-6. In this study, XJZT and JJS were significantly decreased the mRNA expression levels of NF-κB, IL-6, IL-8, and TNF-α, but remarkably increased the IL-10 mRNA expression. Furthermore, the results showed that XJZT and JJS could decrease the protein levels of IκB-α and NF-κB, and remarkably decrease the p-IκB-α and p-NF-κB protein levels in liver. These results illustrated that the XJZT and JJS inhibited the production of pro-inflammatory mediators in livers through down-regulating the NF-κB signal pathway.

## Conclusion

Our data confirmed that 10 g/kg XJZT and 3 g/kg JJS prescriptions exhibited strong hepatoprotective effect on the liver of piglets, which have a strong relationship with their anti-oxidative and anti-inflammatory capabilities by activating the Nrf2/NF-κB pathway. Accordingly, 10 g/kg XJZT and 3 g/kg JJS prescriptions can act as potential materials for drug and functional food development to prevent oxidative stress and inflammatory of piglets.

## Data availability statement

The original contributions presented in the study are included in the article/supplementary material, further inquiries can be directed to the corresponding author.

## Ethics statement

The animal study was reviewed and approved by the Experimental Animal Care and Use Committee of Jiangxi Agricultural University.

## Author contributions

XW, YW, and FY contributed to conception and design of the study. YM, AH, and TX organized the database. FW, GZ, and YY performed the statistical analysis. HC wrote the first draft of the manuscript. XG, AH, and TX wrote sections of the manuscript. All authors contributed to manuscript revision, read, and approved the submitted version.

## Funding

This work was supported by Major Research and Development Projects of Jiangxi Province (20194ABC28008) and Spirit Jinyu Biological Pharmaceutical Co., Ltd. (Huhhot, Inner Mongolia, China). The authors declare that this study received funding from Spirit Jinyu Biological Pharmaceutical Co. Ltd. The funder was not involved in the study design, collection, analysis, interpretation of data, the writing of this article, or the decision to submit it for publication.

## Conflict of interest

The authors declare that the research was conducted in the absence of any commercial or financial relationships that could be construed as a potential conflict of interest.

## Publisher's note

All claims expressed in this article are solely those of the authors and do not necessarily represent those of their affiliated organizations, or those of the publisher, the editors and the reviewers. Any product that may be evaluated in this article, or claim that may be made by its manufacturer, is not guaranteed or endorsed by the publisher.

## References

[1] Martínez-Miró S Tecles F Ramón M Escribano D Hernández F Madrid J. Causes, consequences and biomarkers of stress in swine: an update. BMC Vet Res. (2016) 12:171. 10.1186/s12917-016-0791-8 27543093 PMC4992232

[2] Barnett JL Hemsworth PH Cronin GM Jongman EC Hutson GDA. review of the welfare issues for sows and piglets in relation to housing. Aust J Agric Res. (2001) 52:1–28. 10.1071/AR00057

[3] Frankič T Salobir J. In vivo antioxidant potential of Sweet chestnut (Castanea sativa Mill) wood extract in young growing pigs exposed to n-3 PUFA-induced oxidative stress. J Sci Food Agric. (2011) 91:1432–9. 10.1002/jsfa.4328 21384375

[4] Bacou E Haurogne K Mignot G Allard M De Beaurepaire L Marchand J. Acute social stress-induced immunomodulation in pigs high and low responders to ACTH. Physiol Behav. (2017) 169:1–8. 10.1016/j.physbeh.2016.11.012 27867043

[5] Cruzen SM Baumgard LH Gabler NK Pearce SC Lonergan SM. Temporal proteomic response to acute heat stress in the porcine muscle sarcoplasm. J Anim Sci. (2017) 95:3961–71. 10.2527/jas.2017.1375 28992025

[6] Pi J Zhang Q Fu J Woods CG Hou Y Corkey BE. ROS signaling, oxidative stress and Nrf2 in pancreatic beta-cell function. Toxicol Appl Pharmacol. (2010) 244:77–83. 10.1016/j.taap.2009.05.025 19501608 PMC2837798

[7] Yin J Wu MM Xiao H Ren WK Duan JL Yang G. Development of an antioxidant system after early weaning in piglets. J Anim Sci. (2014) 92:612–9. 10.2527/jas.2013-6986 24352957

[8] Jang KB Kim SW. Supplemental effects of dietary nucleotides on intestinal health and growth performance of newly weaned pigs. J Anim Sci. (2019) 97:4875–82. 10.1093/jas/skz334 31665463 PMC6915224

[9] Tsai WH Yang CC Li PC Chen WC Chien CT. Therapeutic potential of traditional chinese medicine on inflammatory diseases. J Tradit Complement Med. (2013) 3:142–51. 10.4103/2225-4110.114898 24716170 PMC3924991

[10] Shingnaisui K Dey T Manna P Kalita J. Therapeutic potentials of Houttuynia cordata Thunb. against inflammation and oxidative stress: A review. J Ethnopharmacol. (2018) 220:35–43. 10.1016/j.jep.2018.03.038 29605674 PMC7127360

[11] He DY Dai SM. Anti-inflammatory and immunomodulatory effects of paeonia lactiflora pall. a traditional chinese herbal medicine. Front Pharmacol. (2011) 2:10. 10.3389/fphar.2011.00010 21687505 PMC3108611

[12] Jiang C Liu P Zhang J Bao W Qiu S Liang Y. Clinical study of effects of jian ji ning, a chinese herbal medicine compound preparation, in treating patients with myasthenia gravis via the regulation of differential MicroRNAs expression in serum. Evid Based Complement Alternat Med. (2014) 2014:518942. 10.1155/2014/518942 24734107 PMC3956408

[13] Zhang YT Xi YL Liu M Ma HB. The study of flavonoids and polysaccharide content and antioxidant capacity *in vitro* of compound chinese herbal medicine in changbai mountain. Adv Mater Res. (2014) 181:1036–9. 10.4028/www.scientific.net/AMR.926-930.1036

[14] Kai L Lei S. From Preventive Treatment theory to discuss xiaojianzhong soup in the treatment of liver disease. Acta Chin Med. (2014) 29:1743–4. 10.16368/j.issn.1674-8999.2014.12.018

[15] Yin X Song F Gong Y Tu X Wang Y Cao S. A systematic review of antibiotic utilization in China. J Antimicrob Chemother. (2013) 68:2445–52. 10.1093/jac/dkt223 23800903

[16] Alshathly MR. Efficacy of Ginger (Zingiber officinale) in ameliorating streptozotocin-induced diabetic liver injury in rats: histological and biochemical studies. J Microsc Ultrastruct. (2019) 7:91–101. 10.4103/JMAU.JMAU_16_19 31293891 PMC6585475

[17] Shao YX Gong Q Qi XM Wang K Wu YG. Paeoniflorin ameliorates macrophage infiltration and activation by inhibiting the TLR4 signaling pathway in diabetic nephropathy. Front Pharmacol. (2019) 10:566. 10.3389/fphar.2019.00566 31191309 PMC6540689

[18] Pietta PG. Flavonoids as antioxidants. J Nat Prod. (2000) 63:1035–42. 10.1021/np9904509 10924197

[19] Edris AE. Pharmaceutical and therapeutic potentials of essential oils and their individual volatile constituents: a review. Phytother Res. (2007) 21:308–23. 10.1002/ptr.2072 17199238

[20] Polycarpo GV Andretta I Kipper M Cruz-Polycarpo VC Dadalt JC Rodrigues PHM. Meta-analytic study of organic acids as an alternative performance-enhancing feed additive to antibiotics for broiler chickens. Poult Sci. (2017) 96:3645–53. 10.3382/ps/pex178 28938776 PMC5850820

[21] Zhang L Hu Y Duan X Tang T Shen Y Hu B. Characterization and antioxidant activities of polysaccharides from thirteen boletus mushrooms. Int J Biol Macromol. (2018) 113:1–7. 10.1016/j.ijbiomac.2018.02.084 29458100

[22] Tian Y Li Z Shen B Zhang Q Feng H. Protective effects of morin on lipopolysaccharide/d-galactosamine-induced acute liver injury by inhibiting TLR4/NF-κB and activating Nrf2/HO-1 signaling pathways. Int Immunopharmacol. (2017) 45:148–55. 10.1016/j.intimp.2017.02.010 28213269

[23] Dai XY Zhao Y Ge J Zhu SY Li MZ Talukder M. Lycopene attenuates di(2-ethylhexyl) phthalate-induced mitophagy in spleen by regulating the sirtuin3-mediated pathway. Food Funct. (2021) 12:4582–90. 10.1039/D0FO03277H 33908429

[24] Dai XY Li XW Zhu SY Li MZ Zhao Y Talukder M. Lycopene Ameliorates Di(2-ethylhexyl) Phthalate-Induced Pyroptosis in Spleen via Suppression of Classic Caspase-1/NLRP3 Pathway. J Agric Food Chem. (2021) 69:1291–9. 10.1021/acs.jafc.0c06534 33475360

[25] Zheng P Yu B He J Yu J Mao X Luo Y. Arginine metabolism and its protective effects on intestinal health and functions in weaned piglets under oxidative stress induced by diquat. Br J Nutr. (2017) 117:1495–502. 10.1017/S0007114517001519 28701241

[26] Novais AK Deschêne K Martel-Kennes Y Roy C Laforest JP Lessard M. Weaning differentially affects mitochondrial function, oxidative stress, inflammation and apoptosis in normal and low birth weight piglets. PLoS ONE. (2021) 16:e0247188. 10.1371/journal.pone.0247188 33606751 PMC7894895

[27] Er J Xian Z Qi S Jin Z Sheng L Guang J. The effect of compound Chinese herbal preparation on muscle development and meat quality of broiler. Jiangsu J Agric Sci. (2018) 34:374–84. 10.3969/j.issn.1000-4440.2018.02.022

[28] Hu H Xu K Wang K Zhang F Bai X. Dissecting the effect of berberine on the intestinal microbiome in the weaned piglets by metagenomic sequencing. Front Microbiol. (2022) 13:862882. 10.3389/fmicb.2022.862882 35464928 PMC9021597

[29] Wang WK Hsu TL Huang ZY Wang YY. Collective effect of a Chinese formula–a study of xiao-jian-zhong-tang. Am J Chinese Med. (1995) 23:299–304. 10.1142/S0192415X95000353 8571926

[30] Yong Z Qian L Bin W. Effect of xiaojianzhong decoction on lipid peroxidation and cyclic nucleotides in rat with deficiency and cold of spleen and stomach. Chin J Exp Tradit Med Formulae. (2011) 17:151–4. 10.13422/j.cnki.syfjx.2011.23.065

[31] Yan T Rui L Xiao W. Alleviating action of jingfang baidu san on acute alcoholism in mise. Chung-kuo Shih Yen Fang Chi Hsueh Tsa Chih. (2011) 17:221–3. 10.13422/j.cnki.syfjx.2011.21.067

[32] El-Demerdash FM El-Sayed RA Abdel-Daim MM. Rosmarinus officinalis essential oil modulates renal toxicity and oxidative stress induced by potassium dichromate in rats. J Trace Elem Med Biol. (2021) 67:126791. 10.1016/j.jtemb.2021.126791 34022565

[33] Zhang L Zhang W Zheng B Tian N. Sinomenine attenuates traumatic spinal cord injury by suppressing oxidative stress and inflammation via Nrf2 pathway. Neurochem Res. (2019) 44:763–75. 10.1007/s11064-018-02706-z 30603983

[34] Guo Z Chen X Huang Z Chen D Yu B Chen H. Dietary dihydromyricetin supplementation enhances antioxidant capacity and improves lipid metabolism in finishing pigs. Food Funct. (2021) 12:6925–35. 10.1039/D0FO03094E 34132271

[35] Wu QJ Wang ZB Wang GY Li YX Qi YX. Effects of feed supplemented with fermented pine needles (Pinus ponderosa) on growth performance and antioxidant status in broilers. Poult Sci. (2015) 94:1138–44. 10.3382/ps/pev013 25834246

[36] Milbury PE Kalt W. Xenobiotic metabolism and berry flavonoid transport across the blood–brain barrier. J Agric Food Chem. (2010) 58:3950–6. 10.1021/jf903529m 20128604

[37] Hou X Zhang J Ahmad H Zhang H Xu Z Wang T. Evaluation of antioxidant activities of ampelopsin and its protective effect in lipopolysaccharide-induced oxidative stress piglets. PLoS ONE. (2014) 9:e108314. 10.1371/journal.pone.0108314 25268121 PMC4182461

[38] Wang R Wang J Song F Li S Yuan Y. Tanshinol ameliorates CCl4-induced liver fibrosis in rats through the regulation of Nrf2/HO-1 and NF-κB/IκBα signaling pathway. Drug Des Devel Ther. (2018) 12:1281–92. 10.2147/DDDT.S159546 29844659 PMC5961642

[39] Kaltschmidt C Greiner JFW Kaltschmidt B. The transcription factor NF-κB in stem cells and development. Cells. (2021) 10:2042. 10.3390/cells10082042 34440811 PMC8391683

[40] Kumar P Sulakhiya K Barua CC Mundhe N. TNF-alpha, IL-6 and IL-10 expressions, responsible for disparity in action of curcumin against cisplatin-induced nephrotoxicity in rats. Mol Cell Biochem. (2017) 431:113–22. 10.1007/s11010-017-2981-5 28258441

[41] Hira K Sajeli Begum A. Methods for evaluation of TNF-α inhibition effect. Methods Mol Biol. (2021) 2248:271–9. 10.1007/978-1-0716-1130-2_21 33185884

