# Corrigendum: The beneficial effects of traditional Chinese medicine on antioxidative status and inflammatory cytokines expression in the liver of piglets

**DOI:** 10.3389/fvets.2022.1063573

**Published:** 2022-11-09

**Authors:** Xiaoyu Wang, Yun Wang, Yaqin Mao, Aiming Hu, Tianfang Xu, Yan Yang, Feibing Wang, Guangbin Zhou, Xiaowang Guo, Huabin Cao, Fan Yang

**Affiliations:** ^1^Jiangxi Provincial Key Laboratory for Animal Health, Institute of Animal Population Health, College of Animal Science and Technology, Jiangxi Agricultural University, Nanchang, China; ^2^Department of Animal Science and Technology, Jiangxi Biotech Vocational College, Nanchang, China; ^3^China Institute of Veterinary Drug Control, MOA Center for Veterinary Drug Evaluation, Beijing, China; ^4^Jian City Livestock and Veterinary Bureau, Ji'an, China; ^5^Jiangxi Agricultural Technology Extension Center, Nanchang, China; ^6^Agricultural Technology Extension Center, Jinxi County Agriculture and Rural Bureau, Fuzhou, China; ^7^Animal Epidemic Prevention and Quarantine Unit, Fengcheng Agricultural and Rural Bureau, Fengcheng, China; ^8^Yichun Agriculture and Rural Affairs Bureau, Yichun, China

**Keywords:** traditional Chinese medicine, piglet, antioxidant capability, inflammation, liver

In the published article, there was an error in affiliation(s) [6]. Instead of “Agricultural Technology Extension Center, Jinxi County Agriculture and Rural Bureau, Fuzhou, China,” it should be “Jiangxi Agricultural Technology Extension Center, Nanchang, China.”

In the published article, there was an error in affiliation(s) [7]. Instead of “Animal Epidemic Prevention and Quarantine Unit, Fengcheng Agricultural and Rural Bureau, Fengcheng, China,” it should be “Agricultural Technology Extension Center, Jinxi County Agriculture and Rural Bureau, Fuzhou, China.”

In the published article, there was an error in affiliation(s) [8 (Guangbin Zhou)]. Instead of “Yichun Agriculture and Rural Affairs Bureau, Yichun, China,” it should be “Animal Epidemic Prevention and Quarantine Unit, Fengcheng Agricultural and Rural Bureau, Fengcheng, China.”

The authors apologize for this error and state that this does not change the scientific conclusions of the article in any way. The original article has been updated.

## Publisher's note

All claims expressed in this article are solely those of the authors and do not necessarily represent those of their affiliated organizations, or those of the publisher, the editors and the reviewers. Any product that may be evaluated in this article, or claim that may be made by its manufacturer, is not guaranteed or endorsed by the publisher.

